# How to Break Through the Ceiling of Scientific Progress?—Innovation Challenges and Pathways Based on the Fitness Landscape Framework

**DOI:** 10.1002/exp2.70225

**Published:** 2026-10-13

**Authors:** Kunjie Zhu, Li Sun, Peng Du

**Affiliations:** ^1^ Institutes of Science and Development Chinese Academy of Sciences Beijing China; ^2^ University of Chinese Academy of Sciences Beijing China; ^3^ Bureau of Planning and Policy National Natural Science Foundation of China Beijing China

**Keywords:** evolutionary innovation, fitness landscape framework, institutional design, interdisciplinary combination, research paradigms, scientific stagnation

## Abstract

Science has not hit an absolute ceiling; it is stranded on a local peak. Viewed through Wright's fitness landscape, today's evaluation and funding systems over‐select, refining work within established paradigms while suppressing the drift and recombination needed to cross fitness valleys. Restoring room for non‐consensus, curiosity‐driven and interdisciplinary research is an institutional task as much as an intellectual one.

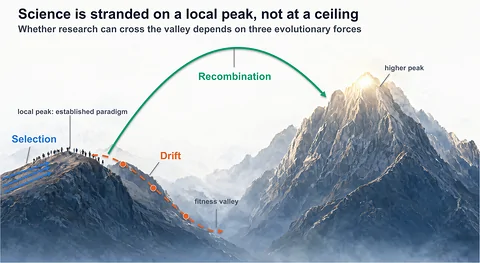

A single pattern is now visible in field after field: research investment has grown sharply, while truly disruptive discoveries have grown rare.

Over the past five decades, the annual volume of global research publications has surged from hundreds of thousands to 3.5 million papers, and annual global R&D expenditure has exceeded $3.4 trillion (2024) [1]. Yet against this expansion, paradigm‐shifting discoveries have become increasingly scarce. A 2023 analysis in *Nature* showed that the CD index, a measure of disruptiveness in papers and patents, has steadily declined from 1945 to 2010 [2], indicating that contemporary research increasingly consolidates existing knowledge rather than opens new frontiers. Nobel Prize trends corroborate the pattern: relative to the explosive growth in researchers and funding, the rate of foundationally paradigm‐shifting awards has lagged, and few post‐1970 discoveries rival the conceptual reach of relativity, quantum mechanics, or the DNA double helix [3]. *The Atlantic* posed the question directly: has science hit a ceiling?

This is not alarmism. Artificial intelligence, often described as the crown jewel of modern technology, shows the same pattern: applications advance at breakneck speed, yet the underlying theory has changed little. The core architectures of deep learning trace back decades, with backpropagation formalized in 1986, convolutional networks demonstrated by LeCun in 1989, and LSTMs introduced in 1997; the attention mechanism underlying transformer models predates the 2017 transformer paper, having been introduced for neural machine translation in 2014. What we are witnessing is engineering refinement and scaling rather than upheaval in scientific principles.

Conventional explanations invoke the “low‐hanging fruit” thesis: the easiest laws have already been discovered. This view is too simple: it treats scientific knowledge as a finite orchard and ignores the dynamics of scientific progress itself. A more productive starting point treats science as a population navigating a rugged landscape.

## 1 Scientific Progress: A Darwinian Process of Natural Selection

This framing is not a casual analogy but follows from a basic feature of science: scientific progress is fundamentally a Darwinian process of natural selection.

In biology, species generate variation through mutation, and the environment selects the fittest while eliminating the unfit. The succession of scientific theories follows a similar logic. Scientists propose diverse hypotheses (variation), and the scientific community filters them through experimental validation, peer review, and practical testing (selection). Theories with superior explanatory and predictive power endure and propagate; those that fail are discarded or marginalized. The Copernican revolution supplanting geocentrism, relativity subsuming and extending Newtonian mechanics, plate tectonics overturning geosyncline theory: each scientific revolution is a struggle for survival among theories. Thomas Kuhn called these “paradigm shifts” in *The Structure of Scientific Revolutions* [4]; at a deeper level, they are natural selection operating in the realm of knowledge.

Darwin's theory of natural selection itself once faced a difficulty. Pure selection could explain how species adapt to their current environment but not how they achieve evolutionary leaps. If every change must be adaptive, how can a population traverse a path that requires temporary loss of fitness to reach a higher state? In 1932, at the Sixth International Congress of Genetics in Ithaca, geneticist Sewall Wright proposed the shifting balance theory, visualized through what he called the adaptive landscape and now commonly termed the fitness landscape [5], to resolve this puzzle.

## 2 Fitness Landscapes: A Framework for Understanding Evolutionary Dilemmas

Wright pictured genotypic space as an undulating multidimensional landscape: the vertical axis is fitness, the horizontal axes are variations in genotype, and evolution is a population climbing this terrain [6]. The model's central insight exposes a limitation of natural selection. Selection acts like gravity, pushing populations uphill by preserving beneficial mutations and eliminating detrimental ones. Once a population reaches a local peak, selection locks it there; any deviation is downhill and is selected against. The population is trapped at a local optimum, unable to reach taller peaks that lie further away.

The landscape's defining trait is ruggedness: not a single peak, but a mosaic of hills and valleys. Wright argued that natural selection alone leads to evolutionary stagnation. Genuine leaps require two further forces. The first is genetic drift, the random fluctuation of allele frequencies in small populations, which can carry a lineage stochastically against the selection gradient, off a local peak and into the surrounding valley. The second is recombination, the chromosomal crossover that reshuffles existing variation and can generate genotypic combinations distant from current ones; we treat recombination as the closest evolutionary counterpart to interdisciplinary synthesis in the analogy below, while noting that Wright's original third force was migration among subpopulations. Acting together, these three forces let species maintain current adaptations while escaping evolutionary dead ends [7]. Subsequent work has refined this picture; high‐dimensional landscapes are now understood to contain extensive neutral ridges and connected networks of near‐optimal genotypes rather than isolated peaks separated by deep valleys [6], but the diagnostic point that selection alone cannot move a population off a local optimum carries forward.

## 3 From Biological Evolution to Knowledge Evolution

Mapping this framework onto science yields close parallels. Roos and Oliver were among the first to import this construct beyond biology, proposing that individuals, communities, and organizations navigate their own knowledge landscapes of potential peaks and valleys, with climbing constrained by identity and organizational interconnectedness and with apparent single peaks resolving, at finer scale, into series of subpeaks [8]. Their treatment targeted organizational learning and strategy and emphasized landscape topography. We extend the framework in three ways: we apply it to the global science‐production system rather than the firm; we mobilize Wright's full trio of selection, genetic drift, and recombination rather than the topography alone; and we translate each force into specific research‐evaluation and funding levers, including a diagnosis of the contemporary imbalance among them.

The competition and replacement of scientific theories mirror natural selection in knowledge space. A theory's fitness is determined by its explanatory power, predictive accuracy, logical coherence, and alignment with societal needs, much as biological fitness depends on survival and reproduction. High‐fitness theories attract more researchers, secure greater resources, and exert broader influence, just as fitter organisms produce more offspring. The community refines a paradigm through detailed studies that elaborate specifics, broaden applications, and resolve anomalies. This is Kuhn's “normal science”, the knowledge population's ongoing uphill ascent on its current peak.

The difficulty is that knowledge landscapes are themselves rugged and multi‐peaked. Once a paradigm is entrenched, the community climbs higher within it, but may be scaling only a local peak rather than the global optimum. Taller summits may lie nearby, separated by a fitness valley; reaching them requires temporarily setting aside established frameworks and accepting theoretical incompleteness and uncertain outcomes, a fitness dip. Just as natural selection confines biological populations to local peaks, evaluation systems, funding mechanisms, and academic cultures lock scientific communities into prevailing paradigms.

## 4 Science's Predicament: Excessive Selection, Insufficient Drift

Returning to science's dilemma, the core problem in modern research systems is an imbalance among the three forces. We over‐emphasize natural selection and leave too little room for genetic drift and recombination.

Contemporary research evaluation functions as an efficient selection engine. Citation counts, impact factors, project completion rates, and patent conversions gauge each study's fitness and allocate resources and careers accordingly. The system excels in uphill phases, helping scientists refine, integrate, and extend within established paradigms. The declining CD index underscores the point: modern research increasingly excels at gap‐filling, consolidating and enhancing what already exists.

This is also the problem. As local peaks are exhausted, the same selection machinery entrenches science within them. Any downhill move, whether challenging foundational assumptions, pursuing fringe directions, or taking on research with uncertain payoffs, is penalized as a fitness decline. Evaluation systems punish failure, funding prioritizes certainty, and academic norms shun risk. Together, these arrangements suppress science's genetic drift: the seemingly useless peripheral explorations and non‐consensus pursuits that could serve as stepping stones across valleys are culled at inception.

At the same time, entrenched disciplinary silos impede genetic recombination. Each field has built its own theoretical traditions, methodological norms, evaluation criteria, and training pipelines. These interlock and reinforce one another, making cross‐disciplinary flows of knowledge difficult. Interdisciplinary initiatives often remain superficial because genuine recombination would disrupt the resource allocation and assessment frameworks that current systems most strongly defend.

## 5 Rebalancing the Three Forces

If the fitness landscape framework holds, the path through the ceiling is clear. We must rebalance the three forces, preserving selection's efficiency while reclaiming space for drift and recombination.

The first task is to safeguard room for genetic drift. In biology, drift preserves neutral variation and stockpiles raw material for future adaptations. Science requires analogous mechanisms, namely tolerance for research that is incongruent with dominant paradigms or lacks immediate application. This means greater funding for non‐consensus, high‐risk projects and genuine tolerance for failed endeavors. As Kenneth Stanley and Joel Lehman argue in *Why Greatness Cannot Be Planned* [9], the pathways to greatness often bear no resemblance to the end goal. Katalin Karikó’s mRNA work, marginalized for two decades before becoming the COVID‐vaccine platform, illustrates how drift‐style persistence eventually crosses a valley. Today's seemingly useless accumulations may prove pivotal for tomorrow's valley crossings.

The second task is to foster recombination‐style knowledge leaps. Major breakthroughs frequently emerge from creative synthesis of existing knowledge. Newtonian mechanics built on Kepler, Galileo, and Descartes; quantum mechanics fused contributions from Planck, Einstein, and Bohr with the matrix and wave formulations of Heisenberg, Schrödinger, Born, and Dirac. Dismantling disciplinary barriers requires more than the usual advocacy for interdisciplinarity, which often proves cosmetic. The aim is to cultivate genuine problem contexts in which different fields collide and recombine while tackling real‐world challenges. Complex problems inherently span disciplines; organizing research around problems rather than boundaries can catalyze long‐distance jumps in knowledge.

The third task is to encourage undirected novelty‐seeking. In artificial intelligence, Lehman and Stanley introduced novelty search algorithms [10] that prioritize novelty alone, retaining solutions with unique behaviors or outcomes rather than optimizing toward a target. In complex domains, this pursuit of difference can outperform goal‐directed approaches. Consistent with the logic of genetic drift, undirected exploration unconstrained by direction or immediate reward accumulates stepping stones and often yields valuable outcomes of its own. Science must allocate space for such work and honor research pursued because it is interesting, allowing curiosity to take precedence over key performance indicators.

## 6 Considerations in the Chinese Context

These dilemmas are global, but China faces them in a distinctive configuration.

China's technological ascent over recent decades has been substantial. Within the fitness landscape framework, these successes stem largely from efficient natural selection, that is, rapid ascent along known paths. This was the right strategy during catch‐up. As China nears the frontier, however, persisting in follow‐the‐leader modes invites bottlenecks because we are approaching the summits of local peaks. Structural frictions such as rigid disciplinary management, evaluation biases toward certain outcomes, and cultural risk aversion jointly compress the space for drift and recombination, reinforcing local‐optimum lock‐in.

The new nationwide mobilization system excels at focused assaults on defined targets; known peaks demand concentrated climbs. When the question shifts to where the next taller peak lies, and science enters unmapped territory, the dynamics change. Frontier uncertainty demands decentralized exploration, diversified trials, and tolerance for seemingly useless variation, mirroring the biological strategies that traverse fitness valleys.

## 7 Conclusion

Acknowledging this does not negate the value of organized research; it argues for a more balanced institutional design that couples concentrated assaults on known challenges with preserved space for drift‐style non‐consensus work and recombination‐style cross‐boundary exploration. We do not claim this imbalance is the sole driver of slowing breakthroughs; demographic, economic, and measurement factors plausibly contribute as well.

Has science reached its limits? The fitness landscape framework suggests not, but it suggests that we may be stranded on a local peak.

This peak has been carefully developed and efficiently exploited, yielding grand edifices of knowledge. Higher ranges may yet lie nearby, hidden beyond unexplored valleys. Reaching them requires not more runners on the same trail but the wisdom of evolution itself: rebalancing selection, drift, and recombination; enduring short‐term fitness dips; accumulating seemingly useless stepping stones; and accepting the uncertainty of undirected exploration.

Science's vitality has never lain in fortifying the known but in engaging the unknown. In this era of abundant resources and scarce breakthroughs, sustaining that vitality has become as much an institutional task as an intellectual one: keeping drift and recombination alive when selection alone would extinguish them.

## Conflicts of Interest

The authors declare no conflict of interest.

## Funding

This work was financially supported by the National Natural Science Foundation of China (L2424120, L2524002).
